# Influencing Factors and Theoretical Models for the Surface Topography in Diamond Turning Process: A Review

**DOI:** 10.3390/mi10050288

**Published:** 2019-04-28

**Authors:** Chunlei He, Wenjun Zong

**Affiliations:** Center for Precision Engineering, Harbin Institute of Technology, Harbin 150001, China; h_chunlei@126.com

**Keywords:** single-point diamond turning, surface topography, tool edge waviness, vibration, work material defect

## Abstract

In this work, the influencing factors and corresponding theoretical models for the surface topography in diamond turning process are reviewed. The surface profile on one tool feed is the elementary unit of surface topography. The influences coupled with the models of the duplication effect of the tool edge profile, material spring back, and plastic side flow are outlined in this part. In light of the surface profile on one tool feed and “trim principle”, the modeling methods of surface topography along the radial direction (2D surface topography) are commented. Moreover, the influence of the vibration between the diamond tool and workpiece on the 2D surface topography is discussed, and the theoretical models are summarized. Finally, the issues for modeling of 3D surface topography, particularly the influences of material defects, are analyzed. According to the state-of-the-art surface topography model of the diamond turned component, future work in this field is therefore predicted.

## 1. Introduction

Single-point diamond turning technology is extensively employed in the advanced manufacturing process, such as the fabrication of optics components and the critical parts in the aerospace technology and clean energy [1,2,3,4]. This technology employs the ultra-sharp diamond tool mounted on the ultra-precision lathe, which is capable of achieving the nanometric surface finish and sub-micron form accuracy at the mean time [5,6,7,8]. The fluid film bearings and high-accurate numerical controller are applied on the lathes to control the relative movements between the diamond tool and the workpiece [9,10,11]. Furthermore, the ambient temperature and the surrounding environment have been strictly controlled to acquire the fine surface finish [12,13]. The above controlling process technologies are applied since they can all affect the final surface topography of the diamond turned components, i.e., the surface topography is the comprehensive result of the above influencing factors. Meanwhile, surface topography also has a direct impact on the functional performance, like the optical functions [14,15,16]. Therefore, it is of great significance to establish an accurate surface topography model for the diamond turned components. 

To fulfil such requirement, recent advances in the surface topography modeling of a diamond turned component is summarized and discussed in this work, which follows the modeling process of surface topography. First, the surface profile model in relation to one feed rate is analyzed and the corresponding models with the influencing factors in this spatial dimensional, such as tool edge waviness, material spring back and plastic side flow are presented. Afterwards, based on the surface topography in one feed rate, the 2D surface topography in the radial direction can be achieved with consideration of vibration between diamond tool and workpiece. Finally, general models for 3D surface topography are acquired and the influencing factors in this spatial dimension, i.e., mechanical properties in relation to the work material are discussed and recommended to be integrated into the surface topography model. Furthermore, challenges and outlooks for the surface topography model of diamond turned components can be achieved according to the above results. 

## 2. Theoretical Models

**Figure 1:**
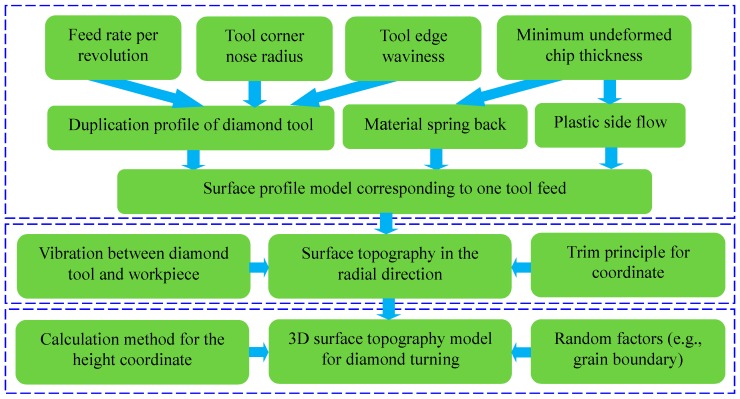
Influencing factors and corresponding issues in the modeling of surface topography.

In this work, three categories of models, i.e., surface profile model in response to one tool feed, surface topography model in the radial direction and 3D surface topography model, will be discussed. The influencing factors and corresponding issues in the modeling process are therefore summarized in Figure 1. The order for these models in Figure 1 is also consistent with the calculation process of surface topography in diamond turning process.

### 2.1. Surface Profile Model Corresponding to One Tool Feed

In diamond turning, the surface topography is generated by the relative motion between the diamond tool and workpiece. Hence, the surface profile model in relation to one feed is the elementary unit of the total surface profile. As revealed by the previous investigations, three main components, i.e., the duplication effect of diamond tool edge profile, material spring back and material plastic side flow, can affect the vertical distance between the highest peak and the lowest valley, i.e., the peak-valley surface roughness *R*_t_ [17,18,19]. Therefore, these three components should be taken into account in the modelling of surface profile corresponding to one tool feed as shown in Figure 1. 

For the duplication effect of diamond tool edge profile, researchers originally employed the circular arc or its simplification form, i.e., the parabola expression as the tool edge profile and established the surface topography model [20,21,22]. On this condition, component in relation to the duplication effect of cutting edge profile can be expressed as

(1) $$R_{\text{tew-wi}}\left(x\right)=r_{\varepsilon }-\sqrt{r_{\varepsilon }^{2}-x^{2}}≅\frac{x^{2}}{2r_{\varepsilon }}\left(-\frac{f}{2}\leq x\leq \frac{f}{2}\right)$$

where *R*_tew-wi_(*x*) is the active tool edge profile in one feed rate (without consideration of the tool edge waviness); *r*_ε_ is the tool corner nose radius; and *f* is the feed rate per revolution. 

**Figure 2:**
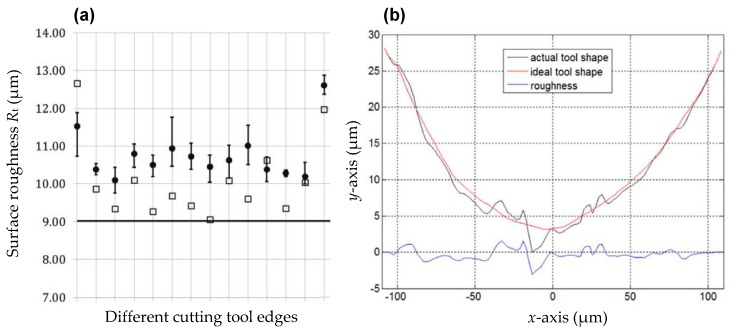
Influence of tool edge waviness on the surface profile: (**a**) Comparisons between the surface roughness results with and without considering tool edge waviness; (**b**) tool edge waviness profile on the diamond tool. Reproduced with permission from [26,29].

For the simplification process, the Taylor formula is applied in the approximation process [15]. However, the disadvantage of this kind of tool edge profile model is also obvious. Due to the state-of-the-art diamond tool fabrication technology [23,24,25], tool edge waviness is inevitably observed on the cutting edge of the diamond tool, which can be measured by the diamond-tool-radius-check (DTRC) system. Due to the existence of tool edge waviness on the cutting tool edge, deviations between the circular arc surface topography and the actual surface topography are unavoidable. For instance, Sung et al. once adopted a high-resolution optical system to capture the image of an active tool nose profile, and their investigations show that the deviation of the tool nose profile, i.e., the tool edge waviness, alone can cause the surface roughness to vary in a large extent [26,27]. As shown in Figure 2a, the hollow square is the simulated peak-valley surface roughness *R*_t_ with consideration of tool edge waviness; and the straight line is the nominal peak-valley surface roughness *R*_t_ = *f*^2^/8*r*_ε_ without consideration of tool edge waviness.

In this work, the tool edge waviness is defined as the deviation between the actual tool shape profile and its corresponding least square circular arc, i.e., the nominal tool shape profile which is expressed in Equation (1). As shown in Figure 2b, the red line (represented as the ideal tool shape) is the nominal tool shape profile, while the black line is the actual tool shape profile. To accurately capture the active part of the diamond tool, He et al. proposed a two-step method, and subsequently integrated it into the 3D surface topography model [28]. Kurniawan et al. employed a replication technique to acquire the actual tool edge profile [29]. Specifically, the tool edge profile is firstly duplicated on a soft material, for instance the aluminium alloy, and then used a 3D non-contact surface profiler to capture the active cutting edge profile. Meanwhile, Zong et al. explored the influence of tool edge waviness in diamond turning of potassium dihydrogen phosphate (KDP) crystals with finite element simulation method, and they pointed out that the tool edge waviness can affect the formation process of cutting chip, which further deteriorates the final achieved surface topography [30]. In addition, Zuo et al. studied the micro/nano texture generation mechanism in double-frequency elliptical vibration diamond cutting, in which the rake face, flank face and tool cutting edge radius are further taken into account [31]. Based on the theoretical and experimental results, they claimed that with consideration of rake face and flank face, the prediction accuracy is obviously improved, which reflects that the influence of rake face and flank face on surface generation is greater in the vibration-assisted diamond turning. 

Childs et al. once studied the dependence of peak-valley surface roughness on the feed rate per revolution with a rounded cutting tool, and reported that under a smaller feed, the tool feature that controls the final surface finish changes from nose radius to cutting edge radius [32,33]. Hence, in addition to the duplication effect to tool edge profile, the influence of cutting edge radius (*r*_n_) should also be taken into account particularly when the feed rate is small enough. Specifically, the influence of cutting edge radius on the surface topography formation is via the existence of the minimum undeformed chip thickness in diamond turning. The theoretical analyses [34,35], molecular dynamic (MD) simulation [36] as well as the experimental observations [37] all reported that there is a linear relationship between the minimum undeformed chip thickness (*h*_Dmin_) and the tool cutting edge radius (*r*_n_), i.e., *h*_Dmin_ = *kr*_n_, where *k* is the corresponding linear coefficient. Further investigations [34] disclosed that the coefficient *k* is in relation to the contact behavior between the diamond tool and workpiece surface, especially the mechanical properties of the work material and the frictional coefficient on the tool-workpiece contact interface. 

**Figure 3:**
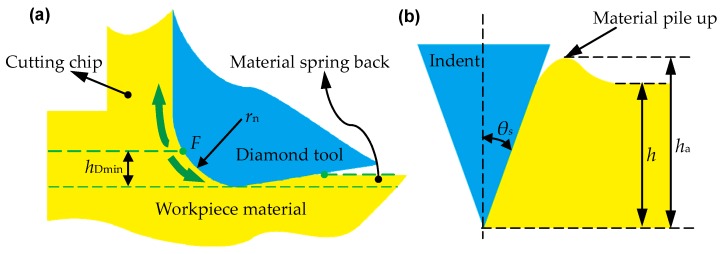
Illustration for the influence of minimum undeformed chip thickness and the calculation model of height of material pile up: (**a**) material removal process in orthogonal plane; (**b**) material pile up *h*_a_ in relation to contact depth *h*. Reproduced with permission from [25,45].

As demonstrated in Figure 3a, due to the existence of the minimum undeformed chip thickness, the work material below the stagnant point *F* undergoes an extensive ploughing and rubbing process, which is a type of plastic deformation without the formation of continuous cutting chips, and further results in some of the work material flowing to the side of the turning mark [38,39]. Subsequently, the phenomena of material plastic side flow and material spring back occur and the machined surface finally forms [40,41]. To calculate the material spring back, Arcona et al. once performed systematic experiments and proposed an empirical model, which demonstrated that the amount of material spring back in metal cutting can be expressed as a proportional function of tool cutting edge radius *r*_n_, the ratio of work material hardness and Yong’s modulus (*H*/*E*) [42,43]. Furthermore, to study the material side flow behavior during the scratch test, Jardret et al. proposed a linear relationship between the height component *h*_a_ induced by the material side flow and the contact depth *h* as shown in Figure 3b, and the expression is given by [44]

(2) $$p_{e}=\frac{h_{a}}{h}=k_{1}\ln\left(\frac{E\cot\theta_{s}}{\sigma_{y}}\right)+k_{2}$$

where *p*_e_ is the elastic recovery rate, i.e., the ratio of *h*_a_ to *h*; *θ_s_* is the semi-apical angle of the indenter; *E* and *σ_y_* are the Young’s modulus and the yield stress of the work material; *k*_1_ and *k*_2_ are the corresponding coefficients acquired from the fitting process, respectively. 

Inspired by the similarities between the scratch test and cutting process, Liu et al. further pointed out that the roughness component (*w*) in relation to the material plastic side flow *w* can be expressed by a function similar to Equation (2), i.e., [45]

(3) $$w=k_{1}\ln\left(\frac{E\cot\theta }{e\sigma_{y}}\right)+k_{2}$$

where *e* is a correct coefficient considering the influence of strain gradient strengthening effect of work material [46]. 

Furthermore, He et al. established the calculation model responsible for the height value in relation to material plastic side flow at the margin point in one feed, which takes feed rate, tool corner nose radius, minimum undeformed chip thickness and effective cutting width into consideration [25]. 

**Figure 4:**
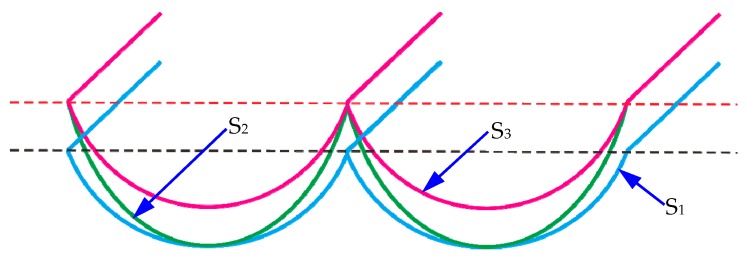
Schematic illustration for the influence of material spring back and plastic side flow on the surface topography in one tool feed (S_1_: only considering the duplication effect of tool edge profile; S_2_: considering the influence of duplication effect and plastic side flow; S_3_: considering the influence of duplication effect, material spring back and plastic side flow). Reproduced with permission from [47].

However, the above solutions only supply the results of material spring back and plastic side flow components in the local position. For instance, in Equation (3), only the height value of material plastic side flow at the margin position is one feed rate is calculated. However, these values are not sufficient for the surface topography model in one feed rate, which means that the distribution functions responsible for the material plastic side flow and material spring back should be supplied. Kong et al. and To et al. studied the material plastic side flow and recovery in diamond turning process [47,48,49], and Kong et al. further pointed out that the effect of material plastic side flow is overwhelmed by the effect of material spring back when the depth of cut is extremely small. Furthermore, the influence of the distribution of the material spring back and plastic side flow on the surface profile in one tool feed is also discussed in [47]. As shown in Figure 4, S_1_ is the surface profile only considering the duplication effect of tool edge profile (including tool edge waviness). Due to the effect of the material plastic side flow, the height of the surface profile S_1_ in one tool feed gradually increases from the center to the margin, and resultantly the surface profile S_2_ forms. Further taking the effect of material spring back into account, the final surface profile on the machined surface can be expressed as S_3_. As demonstrated in [25,45], the value of the material plastic side flow obtains the maximum value at the margin point in one feed rate; this is due to the fact that the work material will flow and accumulate at the side part of the active cutting edge under the high pressure on the tool/workpiece interface. Meanwhile, the value of material spring back reaches the maximum value at the center part in one feed rate. 

Furthermore, Xu et al. carried out molecular dynamics (MD) simulation to investigate the respective influence of material spring back and plastic side flow on surface topography [50]. Their theoretical results show that the height of material spring back almost decreases to zero at the two sides and increases to the maximum value at the center in one feed rate, which is consistent with the model in [47]. Kishawy et al. employed the 3D thermo-viscoplastic finite element simulation to explore the formation of plastic side flow and validated through cutting experiments [51,52]. They reported that larger tool corner nose radius and lower feed rate are the favourable conditions for the generation of material plastic side flow. Meanwhile, Chen et al. adopted the Hertz contact theory coupled with the Johnson-Cook material constitutive model to establish the calculation model for the height component of material plastic side flow, and further verified this model by finite element simulation and actual cutting experiments [53,54]. Summarizing these theoretical results, He et al. further proposed that the general distribution of material spring back and plastic side flow in one feed rate can be approximately calculated using a quadratic function model [28]. The corresponding boundary conditions responsible for the two influencing factors are also established, and on this condition, the surface profile *F*(*x*) in one feed rate can be expressed as

(4) $$F\left(x\right)=R_{\mathrm{tew}}\left(x\right)+\frac{4\left(w_{r}-s_{r}\right)}{f^{2}}\left(-\frac{f}{2}\leq x\leq \frac{f}{2}\right)$$

where *R*_tew_(*x*) is the surface profile in one feed rate with consideration of tool edge waviness; *w*_r_ and *s*_r_ are the values of material plastic side flow in the center (*x* = 0) and material spring back in the margin position (*x* = ±*f*/2) in one feed rate, respectively. 

**Figure 5:**
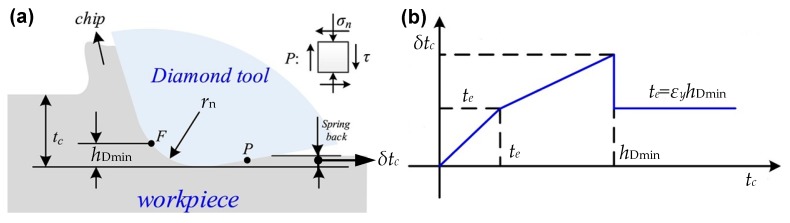
Piecewise function for the distribution of material spring back component in one feed rate: (**a**) illustration for the material spring back in diamond turning process; (**b**) three-section piecewise function form proposed by Zhu *et al*. Reproduced with permission from [55].

Furthermore, due to the insufficient knowledge of calculation method for the height component of material plastic side flow, some researchers attempt to establish the distribution function with respect to the height component of material spring back and apply it to describe the deviation between the kinematic components (*R*_tew_(*x*)) and the final surface profile in one feed rate (*F*(*x*)). In fact, this function can be regarded as the general function as discussed in [28]. For instance, in the fabrication of the micro-freeform lens array, to accurately determine the process parameters required to generate the pre-designed surfaces, Zhu et al. adopt a piecewise function to express the distribution of spring back in relation to the minimum deformed chip thickness (*h*_Dmin_) as demonstrated in Figure 5 [55]. Furthermore, Huang et al. designed a linear function to analyze the amount of material spring back when the undeformed chip thickness ranges from minimum undeformed chip thickness to the maximum chip thickness [56]. Liu et al. claimed that when the undeformed thickness is larger than the minimum undeformed chip thickness, the material spring back amount is zero and hence established the distribution function for material spring back [57]. As an alternative, Guo et al. assumed that when the chip thickness is greater than the minimum undeformed chip thickness, the elastic recovery linearly decrease to a constant value which is in relation to material mechanical properties and cutting edge radius (*r*_n_), and a more complex piecewise function for material spring back is therefore established [58]. 

In diamond turning, the combined effect of plastic side flow, burnishing in relation to the flank face and material spring back is defined as the swelling effect. Researchers further defined the swelling ratio as the proportion between the actual height of measured surface and its nominal height, which can also be employed in the modelling of surface topography. For instance, Cheung et al. defined the local swelling ratio *SP_i_* at the *i*th radial section as the square root of the ratio of the power spectral density for the measured surface profile in one feed rate and the nominal surface roughness spectrum [59], which can be acquired from the nominal surface profile expressed in Equation (1). Similarly, Chen et al. reported that the average swelling ratio could be determined as the ratio of the average height of the measured surface profile to the height of nominal surface profile [60]. Meanwhile, their investigations all demonstrated that the swelling ratio is easily affected by the spindle speed in the actual cutting experiments. Herein, the corresponding theoretical results for the material spring back function are summarized in Table 1. 

However, up until now, the above distribution functions for the material spring back and plastic side flow mainly established on some assumptions and no directly actual experimental observations have been performed to support these assumptions. For the theoretical method, only a few investigations, for instance the molecular dynamic simulation, are performed to analyze the generation of the two influencing factors. Hence, in the future study, the combination of theoretical simulation and experimental observation should be simultaneously employed in the analysis of height component in relation to the material spring back and plastic side flow. Furthermore, in the current investigations, the cross-section shape of the tool edge profile is usually fitted by a circular arc to obtain the cutting edge radius *r*_n_. In fact, Xu et al. has theoretically noted that the shape of the cutting edge shape also affects the stagnation region, chip formation and cutting forces, etc., which should be also further clarified by the actual cutting experiments [61]. 

### 2.2. Surface Topography in the Radial Direction

**Figure 6:**
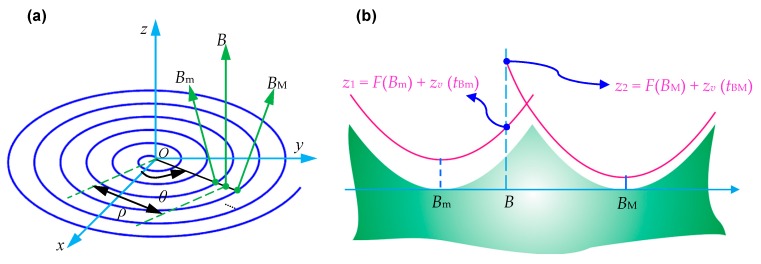
Illustration for the ‘trim principle’ employed in the calculation of surface topography in radial direction: (**a**) polar coordinate of the workpiece surface and position of *B*, *B*_m_ and *B*_M_; (**b**) sectional view of height coordinate of point *B*. Reproduced with permission from [28].

With the theoretical results in Section 2.1, researchers can acquire the surface profile in one feed rate. The following procedure is to establish the surface topography in the radial direction since it is the foundation of the 3D topography model. To obtain the surface topography model, the ‘trim principle’ is extensively employed in the calculation process [62,63,64,65]. As demonstrated in Figure 6a [28], the blue curve is the cutting tool trace in the diamond turning process, and the height coordinate at point *B* in the radial direction *θ* should be calculated. In fact, two or more diamond tool profiles will sweep this point in the diamond turning process and therefore leave their surface profiles, which are represented as the corresponding red arcs as depicted in Figure 6b and the tool tip are at the relevant position *B*_m_ and *B*_M_, respectively. 

The ‘trim principle’ is that the final height coordinate at point *B* is the minimum value among all these height coordinates induced by these tool profiles duplication. More precisely, on the current configurations in Figure 6b, the height coordinate at point *B* is given by

(5) $$z_{B}=\min\left\{F\left(B_{m}\right)+z_{v}\left(t_{\mathrm{Bm}}\right),F\left(B_{M}\right)+z_{v}\left(t_{\mathrm{BM}}\right)\right\}$$

where *F*(*B*_m_) and *F*(*B*_M_) are the height coordinates of point *B* when the tool tip locates respectively at point *B*_m_ and *B*_M_, which can be acquired from the theoretical models discussed in Section 2.1; *z*_v_(*t*) is the vibration component at the cutting time *t*, which will be systematically studied in the this section; *t*_Bm_ and *t*_BM_ are the corresponding times points when the diamond tool locates at the two points.

For the surface topography in the radial direction, researchers have made great effort on the influence of vibration, and for simplification, the vibration is considered as the relative movement between the diamond tool tip and the workpiece surface [66,67]. According to the specific expressions of the vibration component, investigations on the vibration in the surface topography model can be divided into three categories, i.e., the mono-frequency vibration, multi-frequency vibration and multi-direction frequency. For the mono- and multi-frequency vibration condition, only the vibration in the height direction, i.e., *z*-axis is discussed since it is the sensitive direction of vibration impact according to the previous findings [68,69]. The influence of mono-frequency vibration is originally studied, and on this condition a ratio *r*_z-n_ between the vibration frequency (*f_z_*) and the spindle rotation frequency (*f*_n_) is always defined as [70,71]

(6) $$r_{z-n}=\frac{f_{z}}{f_{n}}=\Delta +\varepsilon$$

where the value of Δ is 0 or the positive integer; *ε* is a decimal fraction and in the range of −0.5 to 0.5. Hence, the phase shift ϕ after one revolution is therefore expressed as [72]

(7) ϕ=2πε

Herein, the relative movement between the diamond tool and workpiece surface in the height direction, i.e., the infeed direction is expressed as a mono-frequency harmonic equation corresponding to the phase shift [73]

(8) $$z_{v}\left(t\right)=A\sin\left(2\pi f_{z}t-\phi \right)$$

where *A* is the amplitude of the mono-frequency vibration. Furthermore, the tool locus projected on the *x-z* plane (the feed direction is always configured as the *x*-axis) can be expressed as in terms of the phase shift (ϕ) and feed rate (*f*) as [74]

(9) $$z_{f}\left(x\right)=A\left[1-\cos\left(\frac{x}{f}\phi \right)\right]$$

**Figure 7:**
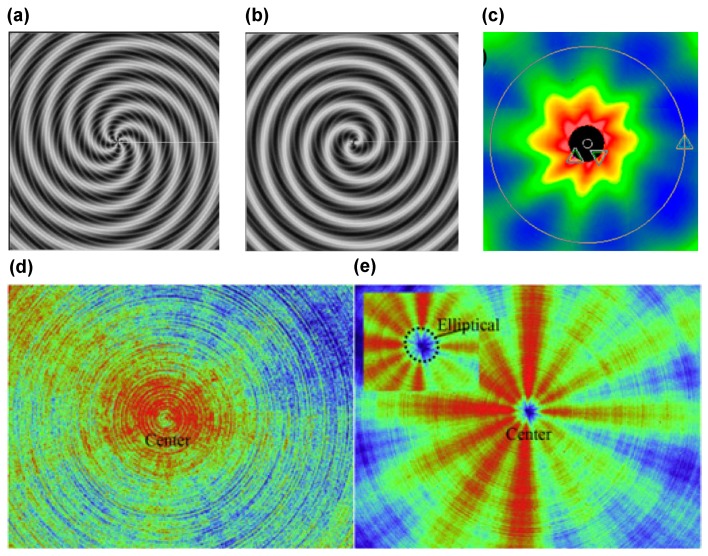
Results of surface topography on mono- and multi-frequency vibration conditions: (**a**) simulation results of surface topography on mono-frequency vibration condition (∆ = 5, *ε* = 0.2); (**b**) simulation results of surface topography on mono-frequency vibration condition (∆ = 2, *ε* = −0.2); (**c**) experimental results on the mono-frequency vibration condition; (**d**,**e**) experimental results on the multi-frequency vibration condition. Reproduced with permission from [75,76,79].

On the mono-frequency vibration condition, regular turning marks in relation to the vibration will be observed on the machined surface. Kim et al. pointed out that the number of the bulge segments is identical to the integer part of the frequency ratio in Equation (6), i.e., the predictions results as demonstrated in Figure 7a,b; while the orientation of the bulge segment depends on the sign of the decimal part *ε* [75]. Specifically, when the value of *ε* is positive the orientation is counter clockwise, and clockwise vice versa as depicted in Figure 7a,b. Tauhiduzzaman et al. experimentally investigated the vibration induced by the imbalance of the spindle, and reported that this kind of vibration is synchronous, which can result in the called ‘spindle star’ that appears as straight concentric spokes as demonstrated in Figure 7c [76]. Zhang et al. further pointed out that regular surface profiles could influence the surface roughness and form error components, which is closely associated with the wavelength of the surface profile component [77]. To reduce the influence of the regular patterns induced by the vibrations, Khanfir et al. developed an active electronic control method with the spindle on magnetic bearings [78]. 

In view of the above disadvantages, recent investigations on the vibration in diamond turning are performed on the multi-direction and multi-frequency conditions. For instance, Zhang et al. proposed a five-degree-of-freedom dynamic model for the aerostatic bearing spindle to explore the mechanism of multi-direction vibration (in the *x*-, *y*- and *z*-direction) and its influence on surface topography in diamond turning [80,81]. Their studies show that on the general effect of the axial and radial vibrations, a spiral and two-fold patterns surface profile generates on the machined surface. Based on the dynamic model, they further pointed out that the surface topography in the center area is mainly affected by the axial vibration of the spindle, while that outside region is primarily affected by the tilting motion of the spindle [82]. In addition, Tian et al. assumed the relative vibration between diamond tool and workpiece in turning direction (infeed direction) and feeding direction to be a simple harmonic motion, and the fine correlations of surface topography between the simulation and experiment results proved the accuracy of the proposed model [83]. Similarly, Huang et al. considered the vibrations in the feed and infeed directions, and established the surface topography model for flat, spherical and freeform surfaces [84,85]. Their investigations indicated that when considering the vibration in feed direction, the tool locus will be more complex and its influence on surface topography cannot be ignored, particularly for the spherical and freeform surfaces. Lin et al. considered the vibrations in three directions, i.e., *x*-, *y*- and *z*-axis, and they claimed that the influence of vibration on surface topography decreases with the decrease of the spindle speed [86]. Furthermore, Qu et al. directly integrated the real-time vibration signals in the feed and infeed directions into the surface topography model in ultra-precision roll die turning and fine consistence is therefore observed [87]. Meanwhile, it should be noted that the original phase of the vibration for [83] to [86] all assumed to be zero for convenience. 

Furthermore, to theoretically study the impact of multi-frequency vibration on the surface topography, He et al. proposed an analytical model for the surface topography considering the influence of multi-frequency vibration, and subsequently performed the corresponding cutting experiments to validate its accuracy [28]. The theoretical results coupled with the experimental observations all demonstrated that the bulge segments on the multi-frequency condition are complex and irregular, which is rather different from the mono-frequency vibration condition. Their theoretical results agree well with the experimental results on the studies of multi-frequency vibration as depicted in Figure 7e. Chen et al. established the finite element model for the machine tool to simulate the multi-mode vibration on the surface generation in diamond fly-cutting process [88,89,90]. They reported that the low-frequency vibration influences the surface roughness error, while the high-frequency vibration affects the figure error in fly-cutting process. Furthermore, Gao et al. pointed out that the fluctuation of the oil source pressure and the flow status of the film of the aerostatic spindle will also affect the figure error in fly-cutting process [91,92]. 

**Figure 8:**
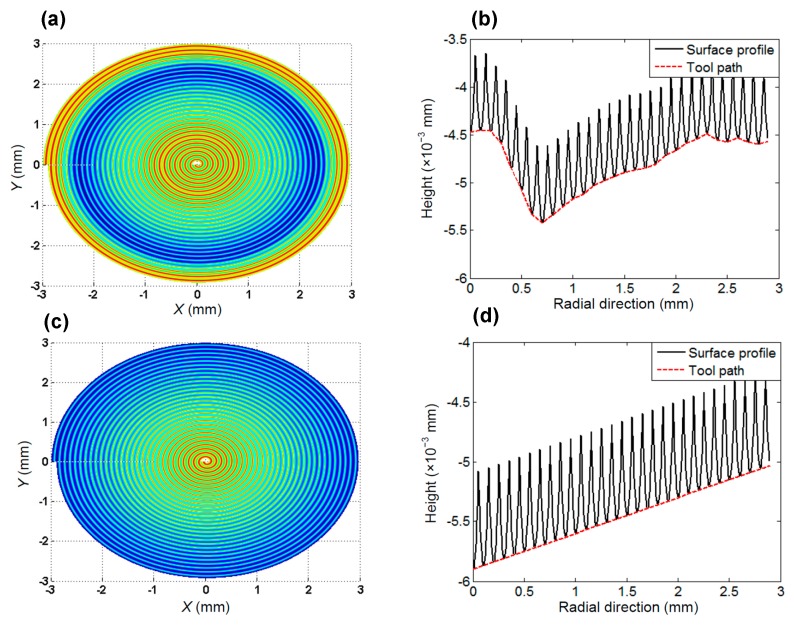
Influence of X-slide carriage straightness error and spindle roll error on the surface topography: (**a**) 2D surface topography when considering the influence of X-slide straightness error; (**b**) sectional view of surface topography corresponding to (**a**); (**c**) 2D surface topography when considering the influence of spindle roll error; (**d**) sectional view of surface topography corresponding to (**c**). Reproduced with permission from [94].

Generally, the vibration between the diamond tool and workpiece surface is one the kinematic errors in the diamond turning process. In the recent studies, researchers tend to integrate other motion errors into the surface topography model. For instance, Kong et al. analyzed the spindle axial motion error, slide motion error and stroke error on the surface generation in fast servo machining [93]. Yang et al. further analyzed the displacement error, angular error and squareness error in the turning process and proposed the surface topography model including these influencing factors [94]. Their theoretical results prove that the slide straightness and angular errors result in the oscillating and tilting surface profile, while the vibrations can cause the segments on the machined surface as demonstrated in Figure 8. Bittner et al. and Wu et al. considered the tilt error between the moving direction of the diamond tool and the spindle axis, and pointed out this error can lead to a conical profile error on the machined surface topography [95,96]. 

The theoretical results for the vibrations and other influencing factors for the surface topography model on the radial direction are therefore summarized in Table 2. As demonstrated, the influence of vibration on the surface topography has been comprehensively considered and analyzed. However, the other influencing factors, for instance the error components in relation to the machine tool structure, are only preliminarily explored. In fact, the influencing factors of surface topography on the radial direction can be categorized into static error component and dynamic error component. The dynamic error component, including the vibration between the diamond tool and workpiece surface, is time-varying; while the static error components, including the positional and angular errors between the spindle, slide and diamond tool, do not vary with time. Therefore, to achieve a more accurate surface topography model, different strategies should be employed for the different error components. Specifically, the dynamics error components should be monitored by the instruments to acquire the real-time signal. In addition, the static error component is accurately determined before the cutting experiments. Subsequently, a more accurate surface topography model is built under the consideration of both the real-time signal and the measured static error components. 

### 2.3. 3D Surface Topography for Diamond Turned Component

Based on the established surface topography model on the radial direction and those reviewed theoretical results, the 3D surface topography can be achieved. For the 3D surface topography model of a diamond turned component, two issues are mainly discussed in the section. The first one is the calculation method for the *z* coordinate on the diamond turned surface under the 3D condition; and the second one is the modeling for the random factors, especially the defects in the work material. For the first issue, researchers extensively employed the numerical calculation method [83,84,85,86,87]. Specifically, the modeling surface is firstly sampled into a finite number of equally spaced sections on the radial and circumferential directions, and the height coordinates of the sampling points are therefore calculated according to the theoretical models on the radial direction as discussed in Section 2.2. For the values of height coordinate in other positions, the fitting method is therefore introduced in the calculation process. However, from this calculation method, only the the coordinates on the intersectional points are the accurate values. Considering these shortcomings, researchers recently started to develop the analytical method for the 3D surface topography model. For instance, He et al. developed an analytical surface topography model on the polar coordinates condition, and then transformed into the Cartesian coordinates condition with corresponding transformation formula [28]. This method can supply the accurate value for each point on the coordinate plane without any fitting calculation process. 

The second issue is the modeling for the random influencing factors in the diamond turning process, especially the crystallographic orientation (single crystal material) and defects (polycrystal material) in the work material. For the single crystal work material, the influence of crystallographic orientation on the surface topography is always analyzed. For instance, Yuan et al. investigated the effect of the crystallographic orientation on the cutting force and surface quality in the diamond turning process, and selected single crystal aluminium and copper as the work material [97]. A micro-plasticity model responsible for the anisotropy phenomenon in diamond turning is therefore developed. To et al. selected three different crystallographic orientations on the single crystal aluminium in diamond turning, and reported that there is a close dependency of surface topography with crystallographic orientation [98]. Similarly, Chen et al. studied the effect of crystallographic orientation of potassium dihydrogen phosphate (KDP) on the finial surface finish, and pointed out that the variation of surface topography is consistent with the variation of crystallographic orientation [99]. To further reveal the underlying mechanism responsible for the dependence of surface topography on the crystallographic orientation, the molecular dynamics (MD) method is usually employed. For instance, Komanduri et al. once conducted the MD simulation to analyze the influence of crystallographic orientation on surface formation of single crystal aluminium in nanometric cutting, and they disclosed the subsurface deformation, dislocations as well as the extent of anisotropy of workpiece material [100,101]. Li et al. explored the influence of pore and second phase particle on the surface finish in diamond turning of single crystal copper with MD simulation, and reported that the existence of pore promotes the slide of dislocation and leads to strong work hardening [102]. Meanwhile, the influence of second phased particle is closely associated with its hardness relative to the work material. Liu et al. fabricated the small diamond tool with focused ion beam (FIB) technology, and further perform nano-cutting experiments on single crystal silicon using a specialized designed instrument with scanning electron microscopy (SEM) online observation [103]. Their experimental results indicated that the cutting-induced amorphous layer on the machined surface is strongly dependent on the depth of cut and cutting edge radius (*r*_n_). As demonstrated, theoretical analysis and experimental observation have performed on the influencing factors of single crystal work material. However, up to now, no suitable surface topography model considering the influence of crystallographic orientation of the single crystal material has been established, which should be further explored in the future investigation. 

**Figure 9:**
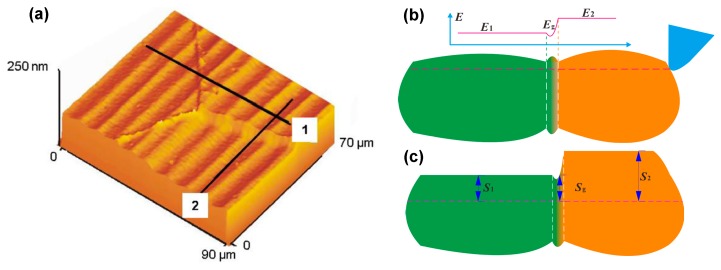
The step structure on the diamond turned of polycrystalline copper and its theoretical explanation: (**a**) the surface topography of step structure on the machined surface; (**b**) surface and the distribution of Young’s modulus before cutting process; (**c**) the step structure formation on the machined surface. Reproduced with permission from [28,107].

Compared with the single crystal material, the polycrystal material is more extensively employed in the diamond turning process, for instance the aluminium alloy and polycrystal copper. In diamond turning, the depth of cut is usually less than the average grain size of polycrystalline material [97]. Moriwaki performed cutting experiments on the machinability of copper and pointed out that the scratches parallel to cutting direction on the diamond turned surface is due to the imperfection on the cutting edge, i.e., tool edge waviness; while the irregular step structure is induced by the grain boundary [104]. Furthermore, the step structure will be strengthened when the surface is machined under a larger depth of cut. Inamura et al. employed a quasi-static method to analyze the nano-cutting process of copper [105]; and they claimed that the formation of surface topography for polycrystalline cooper depends on its crystal orientation. For the formation process of surface topography, they found that the plastic deformation firstly occurs along the grain boundary and then propagates into each grain. Brinksmeier et al. studied the step structure phenomenon and its height value in diamond turning of polycrystalline copper as shown in Figure 9a, and pointed out that the step structure between two grains derives from the variation of elastic recovery [106,107]. They further pointed out that the grain structure of metal substrate is the ultimate limit to the achievable surface roughness. Liu et al. explored the formation mechanism of the step structure for polycrystalline copper, and claimed that the misalignment in the slip direction between the sub-grains and the original grains results in the step structure [108]. To explain the step structure on the diamond turned surface of polycrystalline copper, a theoretical formula for the value of material spring back in diamond turning is developed in [28], and the formation of step structure on the machined surface is reported to heavily depend on the Young’s modulus of the neighbouring grain and their grain boundary. Specifically, as demonstrated in Figure 9b for polycrystalline copper, the Young’s modulus of the two neighbouring grains are different and both are larger than its value on the grain boundary, which finally leads to the step structure as depicted in Figure 9c. Furthermore, to reduce the influence of grain boundary of polycrystalline copper on the final surface finish, Zhang et al. employed the cold-deformation recrystallization annealing method, which has been proved to effectively improve the final surface roughness [109].

In addition to the grain boundary, other kinds of defects can also be observed in the matrix of alloy materials and composites materials. For instance, Ge et al. studied the surface quality when diamond turning of SiC_p_/Al composites, and observed many defects such as pits, voids, micro-cracks, grooves and matrix tearing on the machined surface [110]. Tauhiduzzaman et al. focused on the influence of microstructure of aluminium alloy Al 6061, including grain boundary density and inclusions, and pointed out that using an ultra-fine grain work material can effectively reduce the influence of material microstructure on surface topography [111]. Han et al. experimentally investigated the formation of defects in diamond turning of polycrystalline copper, and pointed out that the dominating defect affecting the formation of surface topography will correspondingly change with the increase of depth of cut [112]. 

**Figure 10:**
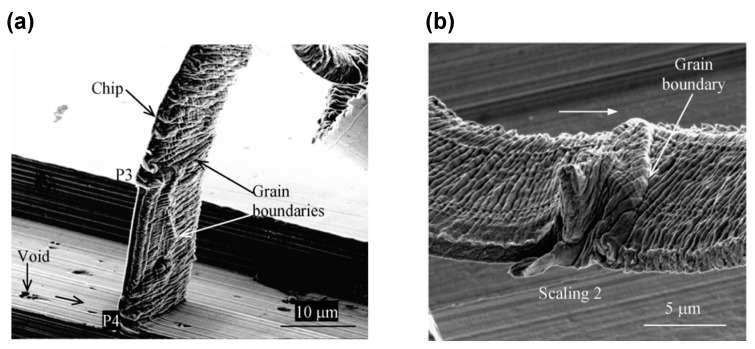
Grain boundary effect on the morphology of cutting chips for different kinds of work material: (**a**) raised line structure on the cutting chip of aluminium alloy Al6061; (**b**) step structure on the cutting chip of polycrystalline copper. Reproduced with permission from [113,114].

To comprehensively reveal the influence of material microstructure on surface topography and analyze its characteristic, Ding et al. conducted orthogonal cutting experiments with a small diamond micro-tool to machine aluminium alloy Al6016 and polycrystalline copper on a vibration-controlled ultraprecision machine tool (Moore Nanotech 350, Moore Nanotechnology Systems, LLC, Swanzey, NH, USA) [113,114]. They found that for the aluminium alloy, the hard particles embedded in the material matrix are brittle, which is responsible for the void and scratch line on the machined surface [113,115]. Furthermore, obvious raised line structure can be observed on the cutting chips as demonstrated in Figure 10a. Meanwhile, for the polycrystalline copper, few hard particles are found on the material matrix, while the grain boundary relevant step structure (not the wrinkled structures on the chip) is clearly observed on the cutting chips in Figure 10b, which is in common with the step structure on the machined surface in Figure 9a. 

**Figure 11:**
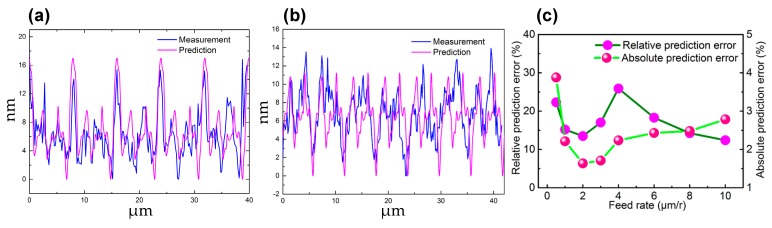
The prediction and measurement results for 2D surface topography on different cutting conditions and the corresponding prediction error: (**a**) topography achieved at *f* = 8 μm/r; (**b**) topography achieved at *f* = 6 μm/r; (**c**) the prediction error vs. feed rate. Reproduced with permission from [28].

To reveal the underlying mechanism for the influence of hard particles, Xu et al. and Sharma et al. performed the molecular dynamics (MD) simulation process, and pointed out that when it encountered the diamond tool cutting edge, the hard particles embedded in the material matrix will be suppressed and subsequently protruded from the generated surface [116,117]. Particles removal or further suppressed into the material matrix are associated with their sizes and locations in the work material. In fact, on different cutting conditions, the dominant factors influencing the surface topography are different. For instance, He et al. once predicted the 2D surface topography of the diamond turned aluminium alloy at different feed rates and compared with the measured results, as shown in Figure 11a,b [28]. Furthermore, the absolute prediction error (the average value for the difference between the prediction and measurement results) and the relative prediction error (the ratio of the absolute prediction error to the actual peak-valley surface roughness *R*_t_ of the surface) were also calculated, as presented in Figure 11c. On a large feed rate condition (*f* = 8 μm/r), the dominant influencing factors on the surface formation are the duplication effect of tool edge profile, material spring back and plastic side flow. Instead, on a small feed rate condition (*f* = 6 μm/r), the influences of material defects, e.g., grain boundary, gradually dominate the formation of the surface topography. The critical impact of material defects on the formation of surface topography on the small feed rate condition (*f* ≤ 2 μm/r) also results in the sharp increase of the absolute and relative prediction errors, as demonstrated in Figure 11c. 

Moreover, in addition to the material property aspects, the ambient environment factors, the configurations of the diamond tool and some other process parameters, such as depth of cut and spindle speed, should also be considered. For instance, Mishra et al. made an experimental investigation and reported that the diamond tool overhang can lead to the excess tool tip vibration and tool wear in machining process, which further affects the final surface topography. The optimized range of the tool overhang, i.e., 12 mm to 16 mm, is recommended through the experimental observations [118,119]. Mir et al. studied the influence of rake angle in diamond turning of silicon with molecular dynamics method, and reported that a positive rake angle diamond tool contributes to the generation of cracks on the machined surface and therefore affects the final surface topography [120]. Meanwhile, the selection of cutting fluids affects the lubrication condition [121], tool wear rate [122] and temperature distribution [123,124] on the contact interface between the diamond tool and workpiece surface, which further has impact on the final surface topography. For the process parameters, Cheung et al. and Zhang et al. once pointed out that the surface roughness decreases with the increase of spindle speed; while the variation trend under different depths of cut is irregular [125,126]. As demonstrated, there are only a few investigations concentrated on ambient environment factors and depth of cut, etc. Unfortunately, no suitable surface topography model with consideration of these influencing factors has been established up to now. 

## 3. Future Work

In this study, the influencing factors of surface topography of the diamond turned components and their corresponding models, as well as the modeling process have been reviewed and summarized. It can be seen that the influence of feed rate, material spring back and the harmonic form vibration between diamond tool and workpiece surface have been comprehensively studied. However, for the other influencing factors, such as tool edge waviness, material plastic side flow, rake angle, grain boundary and other defects in the work material matrix, the quantitative theoretical models are only preliminarily established. In terms of the current findings, we think that some essential future works need to attract more attention. 

Firstly, as revealed in [118], there are ‘uncontrollable parameters’ in the diamond turning process, which include the material defects, tool wear and lubrication condition, etc. Their main characteristic is the random property which results in that it is rather hard to theoretically model their effects. Therefore, in the practical application, it is suggested to introduce the probability method to analyze their effects. For instance, defects such as voids, grain boundary and hard particles are extensively observed in the aluminium alloy material matrix; He et al. employed the uniform distribution coupled with Kolmogorov-Smirnov test method to model the distribution of additional heights in relation to material defects, which effectively improve the prediction accuracy of 3D surface topography model [28]. Furthermore, for the single-crystal work material, the influences of grain orientation and rake angle are distinct and should be further studied and taken into account in the future surface topography model. 

Secondly, in terms of the vibrations between the diamond tool and workpiece surface, their amplitudes and frequencies are always from the Fourier transformation of the original displacement signal and some of the weaker components will be omitted in the calculation process. However, to establish a more accurate surface topography model, the real-time vibration signal is recommended to be integrated into the surface topography modeling process. Meanwhile, the error components in relation to the machine tool structure and diamond tool configurations (for instance, tool overhang) deserve more attention in the topography modeling. 

Thirdly, in the current findings, researchers focused on the various kinds of influencing factors and further integrated them into the surface topography model. In fact, the final surface topography can be regarded as the comprehensive results of these influencing factors. When the impact of one influencing factor is superior to others, corresponding results will be reflected in the machined surface topography. Hence, to inversely infer the dominant influencing factors based on the results of surface topography acquired in experiment should also be given enough attention. 

Fourthly, the quantitative validation of the surface topography model, especially the 3D surface topography model should also be particularly concerned in the practical application. The predication accuracy of the surface topography model is always evaluated by the difference between the measured surface topography and the simulated result. However, it is such to accurately match the corresponding points between the two profiles. To solve this problem, some researchers [28,96] attempted to employ the 2D surface topography, i.e., the radial or circumferential surface profile to evaluate the prediction accuracy, which can be regarded as a local validation method. Meanwhile, some other researchers [85,86,87] compare the surface roughness between the two profiles to validate the accuracy. However, this method is also not comprehensive since two different surface profiles may have the same surface roughness values. Hence, the global evaluation for the prediction accuracy of the 3D surface topography model should also be further explored. 

## 4. Conclusions

This work aims to give a comprehensive understanding of the influencing factors and corresponding modeling methods for the surface topography of the diamond turned component and discover the future developments in the related field. Based on the excellent achievements outlined on the above parts, some conclusions can be drawn as follows

(1) For the surface profile model corresponding to one feed, in addition to the known factors, such as feed rate per revolution and tool corner nose radius, the tool edge waviness and material dependent factors including the material spring back and material plastic side flow should also be given enough attention. Furthermore, the influence mechanism and the corresponding modeling methods of the other factors, such as depth of cut and spindle speed, are deserved to further clarified and subsequently integrated into the surface topography model.
(2) For the surface topography in the radial direction, the ‘trim principle’ is extensively employed in the calculation of the coordinate values in the height direction. Furthermore, researchers have made great effort on the impact of vibration between diamond tool and workpiece, and the expression of harmonic vibration develops from the mono-frequency vibration to the multi-frequency vibration and multi-direction vibration. Herein, to improve the prediction accuracy, the real-time vibration signal and error components related to machine tool as well as cutting tool are recommended to be cooperated into the future surface topography model.
(3) Defects in the work material matrix have a great impact on the final achieved surface topography in diamond turning process, which is closely associated with work material solidification process. For the single crystal material, the influence of the grain orientation should be further taken into account. For the polycrystalline work material, different experimental results are observed for the aluminium alloy and copper material when considering the grain boundary. Hard particles within the polycrystalline work material are responsible for the scratches and raised structures on the machined surface. Furthermore, influencing factors in relation to the ambient environment and the validation for the prediction accuracy should also be concerned on the surface topography model.

## Figures and Tables

**Table 1:** The distribution model of material spring back and/or plastic side flow.

Authors	Expression	Illustration
He et al. [28]	$\delta t_{c}=\frac{4\left(w_{r}-s_{r}\right)}{f^{2}}$	*w*_r_ and *s*_r_ are the values of material plastic side flow and material spring back, respectively.
Zhu et al. [55]	$\delta t_{c}=\{\begin{array}{cc} t_{c} & t_{c}<t_{e}=\varepsilon_{y}h_{D\min}\\ \frac{\left(1-\varepsilon_{p}h_{D\min}\right)-t_{e}}{h_{D\min}-t_{e}} & t_{c}\in \left[t_{e},h_{D\min}\right]\\ t_{e} & t_{c}>h_{D\min} \end{array}$	*t*_e_ is the elastic deformation limit of the work material; *t*_c_ is the chip thickness and *h*_Dmin_ is the minimum undeformed chip thickness.
Huang et al. [56]	$\delta t_{c}=\{\begin{array}{cc} t_{c} & 0\leq t_{c}<t_{c\min}\\ t_{c\min}\frac{t_{c\max}-t_{c\min}}{t_{c\max}-t_{c\min}} & t_{c\min}\leq t_{c}<t_{c\max}\\ 0 & t_{c}>t_{c\max} \end{array}$	*t*_cmax_ and *t*_cmin_ are the maximum and minimum undeformed chip thickness, i.e. *t*_cmin_ = *h*_Dmin_.
Liu et al. [57]	$\delta t_{c}=\{\begin{array}{cc} t_{c} & 0\leq t_{c}<t_{e}\\ p_{e}t_{c} & t_{e}\leq t_{c}<t_{c\min}\\ 0 & t_{c}\geq t_{c\min} \end{array}$	*p*_e_ is the elastic recovery rate when ploughing/rubbing occurs.
Guo et al. [58]	$\delta t_{c}=\{\begin{array}{cc} t_{c} & t_{c}\leq t_{e}\\ p_{e}\left(t_{c}-t_{e}\right)+t_{e} & t_{\mathrm{ce}}\leq t_{c}<t_{c\min}\\ \eta \left(t_{c\max}-t_{c}\right)+t_{e} & t_{c\min}\leq t_{c}<t_{\mathrm{cmax}}\\ t_{e} & t_{c}\geq t_{c\max} \end{array}$	*p*_e_ is the elastic recovery rate of the work material; *η* is the corresponding linear coefficient and determined by *t*_cmax_, *t*_cmin_ and *p*_e_.
Chen et al. [60]	$F\left(x\right)=SP\cdot R_{\mathrm{tew}}\left(x\right)$	*SP* is the swelling ratio.

**Table 2:** Theoretical results for the vibrations employed in the surface topography modelling process.

Authors	Expression	Illustration for the *∅*
Yang et al. [73]	$z_{v}\left(t\right)=A\sin\left(2\pi f_{z}t-\phi \right)$	*∅* is the original phase of the vibration and its value is configured as the phase shift between two adjacent vibrations.
He et al. [28]	$z_{v}\left(t\right)=\sum_{k=1}^{n}A_{k}\sin\left(2\pi f_{k}t+\phi_{k}\right)$	*∅_k_* is the original phase of the *k*th vibration and its value comes from the actual measurement
Tian et al. [83]	$\{\begin{array}{c} x_{v}\left(t\right)=A_{x}\left[1-\cos\left(2\pi f_{x}t+\phi_{x}\right)\right]\\ z_{v}\left(t\right)=A_{z}\left[1-\cos\left(2\pi f_{z}t+\phi_{z}\right)\right] \end{array}$	*∅_x_* and *∅_z_* are assumed to be zero in the modelling process
Huang et al. [84]	$\{\begin{array}{c} x_{v}\left(t\right)=A_{x}\sin\left(2\pi f_{x}t-\phi_{x}\right)\\ z_{v}\left(t\right)=A_{z}\sin\left(2\pi f_{z}t-\phi_{z}\right) \end{array}$	*∅_x_* and *∅_z_* are assumed to be zero in the modelling process
Lin et al. [87]	$\{\begin{array}{c} x_{v}\left(t\right)=A_{x}\sin\left(2\pi f_{x}t-\phi_{x}\right)\\ y_{v}\left(t\right)=A_{y}\sin\left(2\pi f_{y}t-\phi_{y}\right)\\ z_{v}\left(t\right)=A_{z}\sin\left(2\pi f_{z}t-\phi_{z}\right) \end{array}$	*∅_x_*, *∅_y_* and *∅_z_* are assumed to be zero in the modelling process

## Nomenclature

*A*	Amplitude of the mono-frequency vibration
*E*	Yong’s modulus of the work material
*F*(*x*)	Surface profile in one feed rate
*H*	Hardness of the work material
*R*_tew_(*x*)	Surface profile in one feed rate with consideration of tool edge waviness
*R*_tew-wi_(*x*)	Active tool edge profile in one feed rate (without consideration of the tool edge waviness)
*SP*	Average swelling ratio in the radial direction
*SR_i_*	Local swelling ratio *SP_i_* at the *i*th radial section
*e*	Correct coefficient considering the influence of strain gradient strengthening effect of work material
*f*	Feed rate per revolution
*f*_n_	Spindle rotation frequency in diamond turning
*f_z_*	Vibration frequency in diamond turning
*h*	Contact depth in the scratch test
*h*_a_	Height component induced by the material side flow
*h*_Dmin_	Minimum undeformed chip thickness
*k*	Corresponding linear coefficient in relation to the contact behavior
*p*_e_	Elastic recovery rate when ploughing/rubbing occurs.
*r*_n_	Cutting edge radius of diamond tool
*r*_z-n_	Ratio of the vibration frequency to the spindle rotation frequency
*r*_ε_	Corner nose radius of diamond tool
*s*_r_	Value of material spring back in the margin position (*x* =±*f*/2) in one feed rate
*t*_Bm_	Time points when the diamond tool locates at the point *B*_m_
*t*_BM_	Time points when the diamond tool locates at the point *B*_M_
*t*_c_	Chip thickness in diamond turning
*t*_cmax_	Maximum undeformed chip thickness
*t*_cmin_	Minimum undeformed chip thickness
*t*_e_	Elastic deformation limit of the work material
*w*	Roughness component in relation to the material plastic side flow
*w*_r_	Value of material plastic side flow in the center (*x* = 0) in one feed rate
*z*_v_(*t*)	Vibration function at the cutting time *t*
Δ	Integer part of the frequency ratio
δ*t*c	Distribution model of material spring back
*ε*	Decimal fraction part of the frequency ratio and in the range of -0.5 to 0.5
*η*	Coefficient in relation to the material spring back
*θ*	Angular coordinate for the point on the workpiece surface
